# Epidemiology of Bovine Mastitis in Cows of Dharwad District

**DOI:** 10.1155/2014/968076

**Published:** 2014-10-29

**Authors:** Mahantesh M. Kurjogi, Basappa B. Kaliwal

**Affiliations:** P. G. Department of Microbiology and Biotechnology, Karnatak University, Dharwad, Karnataka 580003, India

## Abstract

Bovine mastitis is very common in cows of both developed and developing countries. The prevalence of clinical and subclinical mastitis (SCM) varies from region to region. Hence, the present study was carried out to determine the prevalence of mastitis using three diagnostic tests by considering different risk factors like age, lactation, breed, season, quarters, and herd. The results showed that surf field mastitis test (SFMT) is the most sensitive test for diagnosis of bovine mastitis, the older age and cows with later part of lactation period were more prone to bovine mastitis, and exotic breeds like *Holstein freshen* (HF) were more susceptible to bovine mastitis. The highest incidence of mastitis was recorded in monsoon season. The prevalence of subclinical and clinical mastitis was more in single and two quarters, respectively, and the rate of bovine mastitis was more in unorganized herds. The study concluded that SCM is directly associated with age, lactation period, and environmental factors of the cow and clinical mastitis is more associated with breed of the cow and environmental conditions.

## 1. Introduction

Mastitis is inflammation of mammary gland affecting all the species of domestic animals and is of great concern to dairy industry. Mastitis is very common in cows of both developed and developing countries. Bovine mastitis can be classified into two types, namely, clinical mastitis and SCM. Clinical mastitis is detected by the changes in physical appearance of milk, swelling, redness, and rise in temperature of udder whereas animals with SCM do not exhibit any gross changes in milk or udder and can be detected only through laboratory tests [1]. The diagnosis of SCM is more problematic since milk appears normal. Early diagnosis of mastitis is vital because changes in the udder tissue take place much earlier before they become apparent. Various methods, based on physical and chemical changes of milk and isolation of organisms, are used for diagnosis of subclinical mastitis [2, 3]. However, the logistic and financial considerations involved with sampling all cows for bacteriological culture have precluded this technique from being widely adopted. Milk culture identifies the presence of mastitis pathogens but does not provide a measure of degree of inflammation associated with infection.

The dairy industry is facing a great setback due to high prevalence and incidence of mastitis in milch animals. SCM affects milk quality and quantity causing great economic loss for producers [4, 5]. Annual losses in dairy industry due to mastitis was approximately 2 billion dollars in USA and 526 million dollars in India, in which subclinical mastitis is responsible for approximately 70% of economic losses [6] as most dairymen and farmers are still unaware of impact of SCM.

Even though research work has been done on various aspects of bovine mastitis among dairy cattle in India, region to region variation on prevalence of both clinical mastitis and SCM has been recorded. Since India is a country of diverse agroclimatological conditions it is important to know the prevalence of bovine mastitis in a particular region for planning proper therapeutic, preventive, and control measure for bovine mastitis.

Diagnosis of bovine mastitis depends on the use of various tests and comparative study of these tests in a particular region is very essential for epidemiological investigations. However, a systematic study involving the comparison of different tests for the diagnosis of SCM in cows is not available in the literature even though they are used routinely as diagnostic tests either alone or in combination. Hence, the study aims to compare three mastitis diagnostic tests for their ability to determine the prevalence of mastitis in cows by considering different risk factors like age, lactation, breed, season, quarters, and herds.

## 2. Materials and Methods

### 2.1. Source of Animals

The study was carried out in Dharwad District of Karnataka, India. 263 cows were utilized in the study. The milk samples of cows from four quarters were aseptically collected separately and tested for the presence of mastitis by using three different tests. The procedure was followed every month for a period of one year. The data pertaining to age, lactation, and breed were recorded. The prevalence of clinical mastitis in cows was determined by examination of changes in the udder, namely, redness, rise in temperature, swelling, hardness of udder, changes in milk colour, and reduction in milk quality and quantity.

### 2.2. Diagnostic Tests Used

The milk samples from each quarter of animal were tested by SFMT [7], sodium lauryl sulphate test (SLST) [8], and white side test (WST) [9] to know type of mastitis. For the diagnoses of SCM the positive reaction to these tests along with the absence of clinical signs was used.

### 2.3. The Prevalence

The prevalence was expressed in percent by using the following formula:

(1) $$\begin{array}{c} \text{Prevalence}\;\left(\%\right)=\frac{\text{Number}\;\text{of}\;\text{animals}\;\text{positive}}{\text{Number}\;\text{of}\;\text{animals}\;\text{tested}}\times 100. \end{array}$$

### 2.4. Test-Wise Prevalence of Subclinical and Clinical Mastitis in Cows

The three simple and rapid chemical tests, namely, SFMT, SLST and WST, were used for the diagnosis of bovine mastitis in cows.

### 2.5. Age-Wise Prevalence of Subclinical and Clinical Mastitis in Cows

Cows aged 3 to 13 years were used to know the age-wise prevalence of mastitis.

### 2.6. Lactation-Wise Prevalence of Subclinical and Clinical Mastitis in Cows

Cows in between 1st and 8th months of lactation period were tested to know the lactation-wise prevalence of mastitis.

### 2.7. Breed-Wise Prevalence of Subclinical and Clinical Mastitis in Cows

The breed-wise prevalence of mastitis was studied by using different breeds like* Holstein friesian*, Jersey, Deoni, and nondescriptive (ND) breeds.

### 2.8. Season-Wise Prevalence of Subclinical and Clinical Mastitis in Cows

Four seasons of the year, namely, winter (November, December, January, and February), summer (March, April, and May) monsoon (June, July, and August), and postmonsoon (September and October) seasons, were considered to know the season-wise prevalence of bovine mastitis.

### 2.9. Quarter-Wise Prevalence of Subclinical and Clinical Mastitis in Cows

The milk samples from each quarter of animal were tested to know the quarter-wise prevalence of mastitis.

### 2.10. Herd-Wise Prevalence of Subclinical and Clinical Mastitis in Cows

Herds were categorized into two types, namely, organized and unorganized herds based on the housing facilities; herds with good ventilation, drainage system, adequate water, and bedding facilities were considered organized herds and herds with poor housing design that were maintained in the open field were considered the unorganized herds.

## 3. Statistical Analysis

The data was statistically analysed to know the age-, lactation-, breed-, season, and herd-wise prevalence of subclinical and clinical mastitis. Comparison of proportions and chi-square test were used to know if statistically significant association existed between the age groups, lactation period, different breeds, different season, and types of herd. For all the analysis performed *P* < 0.05 was taken as statistically significant [10].

## 4. Results

### 4.1. Test-Wise Prevalence of Bovine Mastitis

The highest prevalence of SCM in cows was recorded in SFMT followed by SLST and the least was recorded in WST. Similarly the highest prevalence of clinical mastitis in cows was recorded by SFMT, followed by SLST and WST (Table 1).

### 4.2. Age-Wise Prevalence of Bovine Mastitis

The highest prevalence of SCM was recorded in the age group of 7–10 years followed by the group of cows with age greater than 10 years and the least was recorded in the age group of 3–6 years when tested with all three diagnostic tests. Similarly the highest prevalence of clinical mastitis was recorded in the group of cows with age greater than 10 years followed by group of 7–10 years and the least was recorded in the age group of 3–6 years when tested with all three diagnostic tests.

The statistical analysis of data showed there was significant effect on age-wise prevalence of subclinical mastitis, whereas there was no significant effect on age-wise prevalence of clinical mastitis in the study area (Table 2).

### 4.3. Lactation-Wise Prevalence of Mastitis

The highest prevalence of SCM in cows detected by SFMT was in the 5th lactation period followed by the 6th, 2nd, 3rd, 1th, 4th, and 7th and the least was in the 8th lactation period. Similarly SLST diagnosis showed the highest prevalence was in the 5th lactation followed by the 2nd, 6th, 4th, 3rd, 1st, and 8th and least was in 7th lactation period. WST also showed highest prevalence of SCM was in the 5th lactation followed by the 6th, 4th, 2nd, 3rd, 1st, the 7th and no records were seen in the 8th lactation period.

The highest prevalence of clinical mastitis in cows detected by SFMT was in the 5th lactation followed by the 6th, 3rd, 4th, 1th, 7th, and 8th and the least was recorded in the 2nd lactation period. Similarly detection of clinical mastitis was more in the 5th lactation followed by the 8th, 6th, 3rd, 7th, 2nd, and 1st and the least was in the 4th lactation with SLST. The highest prevalence of clinical mastitis detected by WST was in the 5th lactation followed by the 7th, 3rd, 6th, 2nd, 1st, and no records were observed in the 4th, 7th, and 8th lactation.

The statistical studies showed there was significant effect on lactation-wise prevalence of SCM, whereas there was no significant effect on lactation-wise prevalence of clinical mastitis in the study area (Table 3).

### 4.4. Breed-Wise Prevalence of Bovine Mastitis

The highest prevalence of SCM and clinical mastitis in cows detected by all three different tests was in HF followed by Jersey, ND, and Deoni.

The statistical studies showed that there was no significant effect on breed-wise prevalence of subclinical mastitis but there was significant effect on breed-wise prevalence of clinical mastitis when tested with SLST and WST, whereas there was no significant effect on breed-wise prevalence of clinical mastitis when tested with SFMT (Table 4).

### 4.5. Season-Wise Prevalence of Bovine Mastitis

The season-wise prevalence of SCM and clinical mastitis in cows detected by all three tests showed the highest prevalence was in monsoon followed by postmonsoon, winter, and summer seasons.

The statistical analysis of data indicates there was significant effect on season-wise prevalence of subclinical mastitis, whereas there was no significant effect on season-wise prevalence of clinical mastitis in the study area (Table 5).

### 4.6. Quarter-Wise Prevalence of Bovine Mastitis

The prevalence of SCM of cows detected by all three tests indicated that highest incidence of bovine mastitis was in single quarter followed by two and four and the least was recorded in the three quarters.

The prevalence of clinical mastitis of cows detected by SFMT indicated that highest incidence of bovine mastitis was involved in two quarters followed by four and one and the least was recorded in the three quarters. The prevalence of clinical mastitis of cows detected by SLST indicated that the highest incidence of clinical mastitis was involved in two quarters followed by four and one and no records were obtained from the three quarters and WST indicated that highest incidence of clinical mastitis was involved in two quarters followed by four and no records were obtained from the single and three quarters (Table 6).

### 4.7. Herd-Wise Prevalence of Bovine Mastitis

The herd-wise prevalence of SCM and clinical mastitis of cows detected by all three tests indicated that incidence of SCM and clinical mastitis in unorganized herds was more when compared with that of organized herds.

The statistical analysis of data showed there was significant effect on herd-wise prevalence of subclinical mastitis but there was no significant effect on herd-wise prevalence of clinical mastitis when tested with SFMT and SLST, whereas there was a significant effect on clinical mastitis in herd-wise prevalence when tested with WST (Table 7).

## 5. Discussion

The present study indicates that SFMT is the most sensitive test which diagnosed highest number of SCM (46%) and clinical mastitis (8%), whereas Said and Abd-El-Malik [11] reported 38.07% cases in buffaloes on the basis of WST and California mastitis test. Hashmi and Muneer [12] have used cultural examination and reported a figure of 44.9% for buffaloes. Rahman et al. [13] reported a prevalence of 59.2 and 36.8% of SCM in cows and buffaloes, respectively, on the basis of direct, indirect, and cultural examination. Hussain et al.[14] documented a prevalence of 33% in cows and 8% in buffaloes with WST. Shah [15] used Ciba-Geigy mastitis test and found 34.48% buffaloes suffered from SCM. Anwar and Chaudhry [16] reported a prevalence of 47.5% in buffaloes after using Strip Cup test, pH test, and WST. However, Tijare et al. [17] recorded the prevalence of SCM in buffaloes was 70.59%, 57.98%, and 32.77% by WST, CMT, and bromothymol blue test, respectively. Kumar and Thakur [18] detected slightly bigger number of clinical mastitis in buffaloes by using CMT (38) than bromothymol blue test (34). The variation in prevalence of SCM observed in present and previous studies may be due to differences in the sensitivity of tests used for the detection of mastitis.

The present study of age-wise prevalence showed that SCM was more in second group (7–10), whereas clinical mastitis was found to be prominent in the age group >10 years. Similar observations were made by Naghmana, Rasool et al., and Pluvinage et al., [19–21]. Rahman et al. [22] reported 57.5% prevalence of mastitis in the age group of higher than 9 years old and 40.1% in the age group of 7 and 8 years. The present findings are fairly similar to the findings of Sharma [23], Bhikane et al., [24], and Kumar and Sharma [25] and Ameh et al. [26] recorded higher prevalence of mastitis in 4–9-year-old cows. Similarly Biffa et al. [27] suggested older cows are at more risk (44.6%) for the incidence of mastitis than younger cows (23.6%). The high prevalence of SCM and increase in milk production during the age group of 7–10 indicate that production of milk is directly proportional to prevalence of SCM and high prevalence of clinical mastitis in the age group of >10 years may be due to decreased immunity of cows and resistance of bacteria to antibiotics that were indiscriminately used for the treatment of mastitis during previous infections [28–31].

A greater predisposition to infection could be the consequence of a number of characteristics associated with lactation period [32]. In the present investigation lactation-wise prevalence of SCM and clinical mastitis in cows showed that highest prevalence of mastitis was in the 5th lactation, which is in agreement with the findings of Rahman et al. [22]. Pluvinge et al.'s [33] study on mastitis reports that incidence of 8.5% in first lactation is 26% greater than or equal to fifth lactation. The possible cause for high rate of mastitis in 5th lactation may be increased milk yield during this period showing direct correlation between milk yield and the prevalence of bovine mastitis.

The present study of breed-wise prevalence indicated that highest incidence was in HF breed. The exotic breeds like Jersey are more susceptible to bovine mastitis than indigenous breeds [28]. Dutta et al. [34] concluded the risk ratio of developing mastitis in Jersey was approximately two times higher than indigenous breeds. Rahman et al. [22] have reported highest prevalence of mastitis in HF followed by Jersey and the least in indigenous breed. Similarly Biffa et al. [27] reported HF cows are affected at higher rate (56.5%) compared with local zebu (30.9%) and Jersey cows (28.9%). The Boran breed is shown to be more likely to be affected by clinical and SCM when compared with that of short horn zebu breed [35]. The difference in breed-wise prevalence of mastitis may be due to the inheritance characters, immunity of the individual breeds, and also habituation of cows to the climatic conditions.

The season variation is an important factor that directly affects the occurrence of mastitis [36, 37]. The present study revealed that high incidence of mastitis was recorded during monsoon season which is in agreement with Shinde et al. [38], Jadhav et al. [39], and Ameh et al.[26]. In USA Olde Riekerink et al. showed the increase in the somatic cell count during the cold seasons [40]. The present and preceding studies indicate that the risk of developing mastitis in monsoon season is more as the conditions are favorable for the proliferation of pathogenic bacteria.

Quarter-wise study of SCM and clinical mastitis showed that highest prevalence was in single and two quarters, respectively, whereas Iqbal and Siddique [28] reported that in most of the animals two quarters were affected (7.9%) followed by one quarter (6.7%). Kumar and Sharma [25] have reported that mastitis involvement was more in single quarter (52.75%). Similarly Singh and Shankar [41] have recorded higher incidence of mastitis for single quarter (17.4%), as compared to two (2.6%), three (0.3%), and four quarters (2.7%). The difference in quarter-wise prevalence of mastitis is probably due to the fact that predisposing factors like injury, defective sphincters, and so forth could vary from quarter to quarter [28].

Housing facilities contribute to the contamination and exposure of teats to environmental pathogens [42, 43]. In the present study rate of bovine mastitis was more in the cows maintained at unorganized herds. Similarly the percentage of mastitis was 59.5% in unorganized farm where floor was wet and soiled [44]. Kivaria et al.[45] have showed scarcity of water as one of the potential risk factors for prevalence of mastitis.

## 6. Conclusion

In the present study SFMT was found to be most sensitive for the diagnosis of bovine mastitis. The age- and lactation-wise prevalence study indicates older age and cows with later part of lactation stage were more susceptible to bovine mastitis. The breed-wise prevalence of bovine mastitis showed the exotic breeds like HF and Jersey were more prone to bovine mastitis than indigenous cows. Season-wise study showed that cows are more sensitive to bovine mastitis during monsoon. The quarter-wise prevalence of bovine mastitis indicated that preparation of teats and udder for milking is poorly practiced in this region, hence, preventive measures like washing of teats with clean water and drying completely before milking, dipping the teats with any sanitizing solution after milking as to be followed which not only helps to reduce infection of individual cow but also controls the spread of pathogenic bacteria to other animals and humans. The study also indicated that cows in organized herds are less exposed to the bovine mastitis.

The current analysis explored the fact that there exists a significant relationship between age of the cow and the subclinical mastitis but there is no significant association between age and clinical mastitis. Similarly significant association exists between lactation period of cow and subclinical mastitis but not showing in clinical mastitis. However there is no significant relationship between breed of the cow and subclinical mastitis but significant association exits between breed of cow and clinical mastitis diagnosed by SLST and WST. Season-wise prevalence analysis indicates that there is a strong association between seasons and the subclinical mastitis but no such association exists between season and the clinical mastitis. The study also indicated that cow herds and subclinical mastitis have high significant association whereas no major association was recorded between herds and clinical mastitis except when diagnosing with WST.

Considering the results of the current investigation it is concluded that subclinical mastitis is directly associated with age, lactation period, and environmental factors of the cow and clinical mastitis is more associated with the breed of the cow and environmental conditions.

The present study specifies that environment factors play a major role in both subclinical and clinical mastitis; therefore it is recommended to maintain hygienic conditions in the herds for controlling the bovine mastitis.

## Figures and Tables

**Table 1:** Comparison of tests for the diagnosis of subclinical and clinical mastitis in cows.

Test type	Total numbers examined	Subclinical		Clinical	
		Positive	Percentage	Positive	Percentage
SFMT	263	121	46.0	21	8
SLST	263	103	39.1	14	5.5
WST	263	92	34.9	12	4.7

**Table 2:** Association of subclinical and clinical bovine mastitis between age groups.

Age	Total numbers examined	Subclinical positive			Clinical positive		
		SFMT	SLST	WST	SFMT	SLST	WST
3–6	94	26	24	19	6	4	3
7–10	125	70	64	60	11	7	7
>10	44	15	15	13	4	4	3
**χ** ^2^ ** values**	263	8.97^*^	15.4^*^	18.91^*^	0.51	1.34	1.06

^*^Significant: *P* < 0.05.

**Table 3:** Lactation-wise prevalence of subclinical and clinical mastitis in cows.

Test type	Total numbers examined	Subclinical		Clinical	
		Positive	Percentage	Positive	Percentage
1st lactation					
SFMT	40	16	40	2	5.0
SLST	40	10	25	1	2.5
WST	40	10	25	1	2.5
2nd lactation					
SFMT	33	16	48.4	1	3
SLST	33	16	48.4	1	3
WST	33	12	36.3	1	3
3rd lactation					
SFMT	67	31	46.2	6	8.9
SLST	67	25	37.3	4	5.9
WST	67	20	29.8	4	5.9
4th lactation					
SFMT	55	22	40	3	5.2
SLST	55	22	40	1	1.8
WST	55	22	40	0	0
5th lactation					
SFMT	30	22	73.3	5	16.6
SLST	30	20	66.6	4	13.3
WST	30	18	60	4	13.3
6th lactation					
SFMT	18	10	55.5	2	11.1
SLST	18	8	44.4	2	11.1
WST	18	8	44.4	1	5.5
7th lactation					
SFMT	12	3	25	1	3.6
SLST	12	1	8.3	1	3.6
WST	12	1	8.3	0	0.0
8th lactation					
SFMT	8	1	12.5	1	3.4
SLST	8	1	12.5	0	0
WST	8	0	0	0	0
*χ* ^2^ values					
SFMT	263	23.06^*^		5.73	
SLST	263	30.79^*^		8.14	
WST	263	20.28^*^		10.6	

^*^Significant: *P* < 0.05.

**Table 4:** Breed-wise prevalence of subclinical and clinical mastitis in cows.

Test type	Total numbers examined	Subclinical		Clinical	
		Positive	Percentage	Positive	Percentage
Nondescriptive					
SFMT	52	21	40.8	2	3.8
SLST	52	17	32.7	1	2.5
WST	52	15	29.4	1	2.5
Deoni					
SFMT	58	21	36.1	1	1.8
SLST	58	16	28.2	0	0
WST	58	16	22.5	0	0
Jersey					
SFMT	69	33	47.8	7	10.1
SLST	69	24	35	4	5.1
WST	69	24	35	2	2.8
*Holstein Friesian *					
SFMT	84	46	54.7	11	13.2
SLST	84	32	38	9	10.7
WST	84	37	44	9	10.7
*χ* ^2^ values					
SFMT	263	5.6		7.73	
SLST	263	1.74		9.28^*^	
WST	263	4.52		11.35^*^	

^*^Significant: *P* < 0.05.

**Table 5:** Season-wise prevalence of subclinical and clinical mastitis.

Test type	Total numbers examined	Subclinical		Clinical	
		Positive	Percentage	Positive	Percentage
Winter					
SFMT	60	26	43.3	8	13
SLST	60	19	31.6	8	13
WST	60	19	31.6	6	10
Summer					
SFMT	78	22	28.2	7	8.9
SLST	78	16	20.5	4	5.1
WST	78	15	19.2	4	5.1
Monsoon					
SFMT	67	42	62.6	15	22.3
SLST	67	37	56.7	12	17.9
WST	67	35	52.2	12	17.9
Postmonsoon					
SFMT	58	32	55.1	9	15.5
SLST	58	30	51.7	9	15.5
WST	58	27	46.5	8	13.7
*χ* ^2^ values					
SFMT	263	19.56^*^		5.26	
SLST	263	23.97^*^		6.18	
WST	263	20.34^*^		6.28	

^*^Significant: *P* < 0.05.

**Table 6:** Quarter-wise prevalence of subclinical and clinical mastitis in Cows.

Test type	Total numbers examined	Subclinical		Clinical	
		Positive	Percentage	Positive	Percentage
One quarter					
SFMT	263	74	28.2	8	3.0
SLST	263	70	26.5	6	2.2
WST	263	68	26.0	0	0
Two quarters					
SFMT	263	34	12.8	22	8.2
SLST	263	34	12.8	16	6.0
WST	263	26	10.0	16	6.0
Three quarters					
SFMT	263	13	5.1	3	1.0
SLST	263	10	3.8	0	0
WST	263	8	3.2	0	0
Four quarters					
SFMT	263	21	8.0	15	5.8
SLST	263	16	6.2	7	2.8
WST	263	16	6.2	3	1.0

**Table 7:** Herd-wise prevalence of subclinical mastitis in cows.

Test type	Total numbers examined	Subclinical		Clinical	
		Positive	Percentage	Positive	Percentage
Organized herds					
SFMT	112	14	12.5	2	1.7
SLST	112	12	10.7	2	1.7
WST	112	12	10.7	2	1.7
Unorganized herds					
SFMT	151	51	33.7	10	6.6
SLST	151	43	28.4	7	4.6
WST	151	37	24.5	6	3.9
*χ* ^2^ values					
SFMT	263	15.66^*^		3.47	
SLST	263	12.27^*^		1.59	
WST	263	8.06^*^		158.4^*^	

^*^Significant: *P* < 0.05.

## References

[1] Reza V. H., Mehran F. M., Majid M. S., Hamid M. (2011). Bacterial pathogens of intramammary infections in Azeri buffaloes of Iran and their antibiogram. *African Journal of Agricultural Research*.

[2] Batra T. R., McAllister A. J. (1984). A comparison of mastitis detection methods in dairy cattle. *Canadian Journal of Animal Science*.

[3] Emanuelson U., Olsson T., Holmberg O. (1987). Comparison of some screening tests for detecting mastitis. *Journal of Dairy Science*.

[4] Swinkels J. M., Hogeveen H., Zadoks R. N. (2005). A partial budget model to estimate economic benefits of lactational treatment of subclinical *Staphylococcus aureus* mastitis. *Journal of Dairy Science*.

[5] Halasa T., Huijps K., Østerås O., Hogeveen H. (2007). Economic effects of bovine mastitis and mastitis management: a review. *Veterinary Quarterly*.

[6] Varshney J. P., Naresh R. (2004). Evaluation of a homeopathic complex in the clinical management of udder diseases of riverine buffaloes. *Homeopathy*.

[7] Muhammad G., Athar M., Shakoor A., Khan M. Z., Rahman F., Ahmad M. T. (1995). Surf field mastitis test: an inexpensive new tool for evaluation of wholesomeness of fresh milk. *Pakistan Journal of Food Sciences*.

[8] Sharma D. K., Jallewar P. K., Sharma K. K. (2010). Antibiogram of bacteria isolated from bovine subclinical mastitis. *Indian Veterinary Journal*.

[9] Murphy J. M., Hanson J. J. (1941). Modified Whiteside test for the detection of chronic bovine mastitis. *Cornell Veterinary*.

[10] Snedecor G. W., Cochran W. G. (1967). *Statistical Methods*.

[11] Said A. H., Abd-El-Malik A. S. (1968). Diagnosis, incidence and treatment of subclinical mastitis in dairy buffaloes. *Journal of Veterinary Science of the United Arab Republic*.

[12] Hashmi H. A., Muneer M. A. (1981). Subclinical mastitis in buffaloes at Lahore. *Pakistan Veterinary Journal*.

[13] Rahman H., Sambyal D. S., Baxi K. K. (1983). Incidence and aetiology of subclinical mastitis in cows and buffaloes in Punjab. *Journal of Research (Punjab Agricultural University)*.

[14] Hussain M., Naeem K., Iqbal N. (1984). Subclinical mastitis in cows and buffaloes: Identification and drug susceptibility of causative organism. *Pakistan Veterinary Journal*.

[15] Shah A. H. (1987). *Bovine subclinical mastitis due to Staphylococcus aureus and its treatment with cloxacillin, rifampicin and their combination [M.S. thesis]*.

[16] Anwar M., Chaudhry A. Q. (1983). Subclinical mastitis in buffaloes around Lahore. *Pakistan Veterinary*.

[17] Tijare D. B., Singh A. K., Chaturvedi V. K., Dhanesar N. S. (1999). Sensitivity of indirect tests in detection of subclinical mastitis in buffaloes. *Indian Veterinary Journal*.

[18] Kumar P., Thakur D. K. (2001). Comparative efficacy of indirect tests for the detection of mastitis in buffaloes. *Indian Veterinary Journal*.

[19] Naghmana S. (1984). *Bacteriological survey of chronic mastitis in cattle and buffaloes [M.S. thesis]*.

[20] Rasool G., Jabbar M. A., Kazmi S. E., Ahmed A. (1985). Incidence of subclinical mastitis in Nili-Ravi buffaloes and Sahiwal cows. *Pakistan Veterinary Journal*.

[21] Pluvinage P., Ducruet T., Josse J., Monicat F. (1990). Factors of risk of milk cows mastitis. Results of the survey. *Environment and Animal Hygiene*.

[22] Rahman M. A., Bhuiyan M. M. U., Kamal M. M., Shamsuddin M. (2009). Prevalence and risk factors of mastitis in dairy cows. *Bangladesh Veterinarian*.

[23] Sharma S. D. (1978). Studies on some aspects of clinical and subclinical bovine mastitis in relation to etiology, diagnosis and therapeutic with a note on blood in milk. *Veterinary Research Bulletin*.

[24] Bhikane A. U., Anantwar L. G., Yadav G. U., Markandeya N. M., Deshmukh Y. T. Incidence of clinical mastitis in ruminants.

[25] Kumar R., Sharma A. (2002). Prevalence, etiology and antibiogram of mastitis in cows and buffaloes in Hisar, Haryana. *Indian Journal of Animal Sciences*.

[26] Ameh J. A., Edgbe-Nwiyi T., Zaria L. T. (1999). Prevalence of bovine mastitis in Maiduguri Borno State, Nigeria. *Veterinarski Arhiv*.

[27] Biffa D., Debela E., Beyene F. (2005). Prevalence and risk factors of mastitis in lactating dairy cows in Southern Ethiopia. *International Journal of Applied Research in Veterinary Medicine*.

[28] Iqbal J., Siddique M. (1999). Some epidemiological aspects of mastitis in cows and biocharecterization of isolated *Staphylococci*. *Pakistan Veterinary Journal*.

[29] Slettbakk T., Jørstad A., Farver T. B., Holmes J. C. (1995). Impact of milking characteristics and morphology of udder and teats on clinical mastitis in first- and second-lactation Norwegian cattle. *Preventive Veterinary Medicine*.

[30] Radostits O. M., Gay C. C., Blood D. C., Hinchcliff K. W. (2000). Mastitis. *Vetrinary Medicine*.

[31] Dulin A. M., Paape M. J., Nickerson S. C. (1988). Comparison of phagocytosis and chemiluminescence by blood and mammary gland neutrophils from multiparous and nulliparous cows. *The American Journal of Veterinary Research*.

[32] Poutrel B. (1982). Susceptibility to mastitis: a review of factors related to the cow. *Annales de Recherches Veterinaires*.

[33] Pluvinge P., Ducruet T., Josse J., Monicat P. (1989). Factors of risk of milk cows mastitis. Results of the survey. *Dairy Science Abstract*.

[34] Dutta J., Rathore B. S., Mullick S. G. (1990). Lactational incidence rate of mastitis in exotic and crossbreed cows. An epidemiology study. *Indian Journal of Veterinary Pathology*.

[35] Karimuribo E. D., Fitzpatrick J. L., Bell C. E. (2006). Clinical and subclinical mastitis in smallholder dairy farms in Tanzania: risk, intervention and knowledge transfer. *Preventive Veterinary Medicine*.

[36] Johnson H. D., Vanjonack W. J. (1976). Effects of environmental and other stressors on blood hormone patterns in lactating animals.. *Journal of dairy science*.

[37] Shathele M. S. (2009). Weather effect on bacterial mastitis in dairy cows. *International Journal of Dairy Science*.

[38] Shinde S. S., kulkarni G. B., Gangane G. K., Degloorkar N. M. Incidence of mastitis in prebhani district of Maharashtra.

[39] Jadhav K. l., Tripathi V. N., Kale M. M. (1995). Incidence and economics and mammary disorders in holstein X sahiwal cross bred cows. *Indian Journal of dairy Science*.

[40] Olde Riekerink R. G. M., Barkema H. W., Stryhn H. (2007). The effect of season on somatic cell count and the incidence of clinical mastitis. *Journal of Dairy Science*.

[41] Singh R., Shankar H. (2002). Occurrence of mastitis in organized farms. *Indian Journal of Comparative Microbiology, Immunology and Infectious Diseases*.

[42] Smith K. L., Hogan J. S. (1993). Environmental mastitis. *Veterinary Clinics of North America: Food Animal Practice*.

[43] Barkema H. W., Schukken Y. H., Lam T. J. G. M., Beiboer M. L., Benedictus G., Brand A. (1999). Management practices associated with the incidence rate of clinical mastitis. *Journal of Dairy Science*.

[44] Hogan J. S., Smith K. L., Todhunter D. A., Schoenberger P. S. (1990). Bacterial counts associated with recycled newspaper bedding. *Journal of Dairy Science*.

[45] Kivaria F. M., Noordhuizen J. P. T. M., Kapaga A. M. (2004). Risk indicators associated with subclinical mastitis in smallholder dairy cows in Tanzania. *Tropical Animal Health and Production*.

